# Compositional Study of Phospholipids from the Dried Big Head and Opossum Shrimp, Mussel, and Sea Cucumber Using ^31^P NMR Spectroscopy: Content and Fatty Acid Composition of Plasmalogen

**DOI:** 10.3390/molecules27196250

**Published:** 2022-09-22

**Authors:** Eun-Sik Hong, Ji-Hyun Kim, Hee-Jin So, Eun-Ah Park, Ye-Lim Park, Jeung-Hee Lee, Jung-Ah Shin, Ki-Teak Lee

**Affiliations:** 1 Department of Food Science and Technology, Chungnam National University, 99 Daehak-ro, Yuseong-gu, Daejeon 34134, Chungcheongnam-do, Korea; 2 Solus Biotech, 10 Suji-ro, Suji-gu, Yongin 16858, Gyeonggi-do, Korea; 3 Department of Human Nutrition, Food and Animal Sciences, University of Hawaii at Manoa, 1955 East-West Road, Honolulu, HI 96822, USA; 4 Department of Food and Nutrition, Daegu University, 201 Daegudae-ro, Gyeonsan-si 38453, Gyeongsangbuk-do, Korea; 5 Department of Food Processing and Distribution, Gangneung-Wonju National University, 7 Jukheon-gil, Gangneung 25457, Gangwon-do, Korea

**Keywords:** phospholipids, plasmalogen, dried marine animal, ^31^P-NMR

## Abstract

Herein, we present a qualitative and quantitative analysis of the compositions of plasmalogens and phospholipids (PLs) in dried big head shrimp (*Solenocera melantho*), opossum shrimp (*Neomysis awatschensis*), mussel (*Mytilus galloprovincialis*), and sea cucumber (*Apostichopus japonicus*). We also analyze the fatty acid composition of the extracted lipids, phosphatidyl choline (PtdCho), and plasmalogen choline (PlsCho) from each sample. In big head shrimp, opossum shrimp, and mussel, phosphatidyl choline (PtdCho) was the most abundant PL at 1677.9, 1603, and 1661.6 mg/100 g of dried sample, respectively, whereas the most abundant PL in sea cucumber was PlsCho (206.9 mg/100 g of dried sample). In all four samples, plasmalogen ethanolamine (PlsEtn) was higher than phosphatidyl ethanolamine (PtdEtn). The content (mg/100 g of dried sample) of PlsCho was highest in mussel (379.0), and it was higher in big head shrimp (262.3) and opossum shrimp (245.6) than sea cucumber (206.9). The contents (mg/100 g of dried sample) of PlsEtn were in the order of mussel (675.4) > big head shrimp (629.5) > opossum shrimp (217.9) > sea cucumber (51.5). For analyzing the fatty acids at the *sn*-2 position of PlsCho, the consecutive treatment with phospholipase A_1_, solid phase extraction, thin-layer chromatography (TLC), and GC-FID were applied. The most abundant fatty acid was eicosapentaenoic acid (EPA, C20:5, n-3) in big head shrimp and sea cucumber, palmitoleic acid (C16:1, n-7) in opossum shrimp, and docosadienoic acid (C22:2, n-6) in mussel.

## 1. Introduction

**Figure 1:**
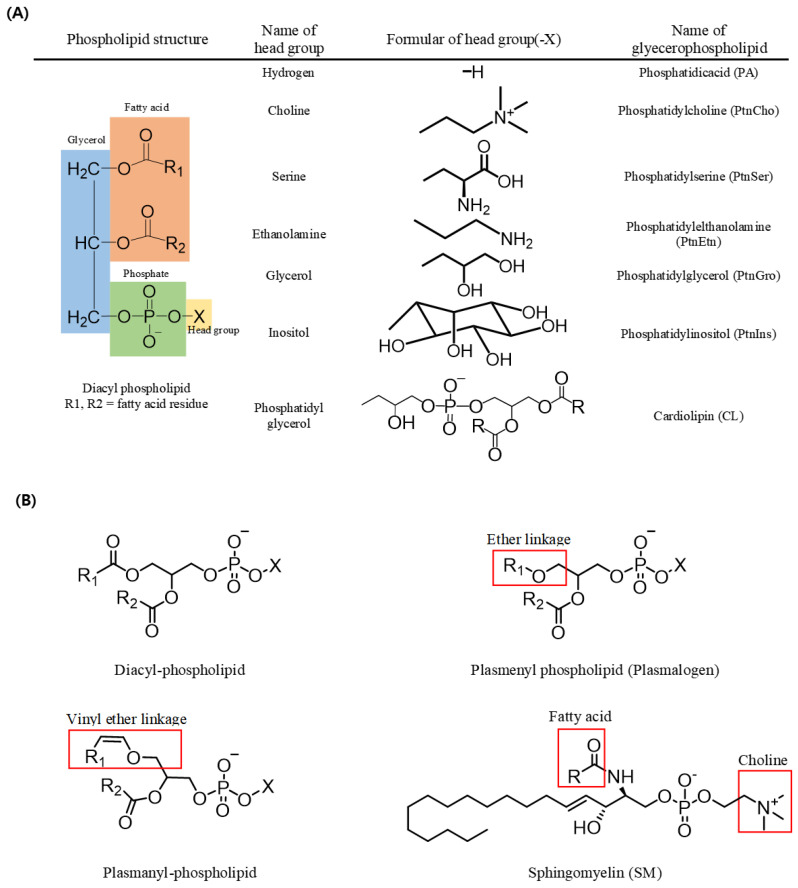
Structures of phospholipids. (**A**) Name of the phospholipid according to the change of the head group in diacyl-phospholipid. (**B**) Structures of diacyl-phospholipid, ether lipid, and sphingolipid.

In triacylglycerols (TAGs, *sn*-1,2,3-triacylglycerols), all three carbons constituting the glycerol backbone are engaged in an ester linkage with a fatty acid molecule, while in most phospholipids (*sn*-1,2-diacylglycerol phospholipids), fatty acids are engaged in ester linkages at the *sn*-1 and *sn*-2 positions and a phosphate and a head group are located at the *sn*-3 position. The type of phospholipid (PL) varies according to the head group; for example, a PL with an ethanolamine head group is phosphatidyl ethanolamine (PtdEtn), whereas that with a choline head group is phosphatidyl choline (PtdCho) (Figure 1). In addition, sphingomyelin (SM) possesses a fatty acid molecule linked with an amine group of the sphingosine backbone along with a phosphocholine in most mammals (Figure 1) [1].

A form of PL with a vinyl group in an ether linkage at the *sn*-1 position of the glycerol backbone is known as a plasmenyl species (i.e., plasmalogen), whereas one with an alkyl group in an ether linkage at the *sn*-1 position is known as plasmanyl species. Plasmalogen is the most widely detected form of PL in vertebrate and invertebrate animals. Such ether-linked PLs also exist in various forms based on the head group linked with the phosphate at the *sn*-3 position, including plasmalogen-ethanolamine (PlsEtn) and plasmalogencholine (PlsCho) (Figure 1) [2,3].

PLs are the main components of the human cell membrane bilayer and play a key role in physiological activities. Among the PLs, plasmalogen is mainly found in the lipid raft of cell membranes enriched with cholesterol. With a vinyl group in an ether linkage at the *sn*-1 position, plasmalogen reinforces the hydrogen bonds among the PL molecules, providing the stability of the cell membrane [4]. A previous study has also shown the correlation between a low content of plasmalogen and certain diseases, such as a correlation between the plasma PlsEtn and the onset of Alzheimer’s disease [5].

In general, high-performance liquid chromatography (HPLC)–evaporative light scattering detection (ELSD) is used for the quantitative analysis of PLs; however, in the case of plasmalogen, the structural similarity with the diacylglycerol phospholipid results in co-elution among the peaks in the HPLC analysis, and thus, simultaneous isolation is difficult, unless there is a combination of successive HPLC separations with different analysis conditions. Hence, the vinyl–ether linkage should be hydrolyzed using an acid or enzyme, and it should be noted that other acyl groups could also be degraded if the acid treatment, which should hydrolyze only vinyl groups, is in excess [6]. In contrast, ^31^P-NMR allows the qualitative and quantitative analyses of plasmalogen and other types of PLs without such pretreatments, as it works on the selectivity (i.e., the variation in chemical shift according to the difference in the electron density around the nucleus) of each PL type. This is useful for the qualitative and quantitative analyses of plasmalogen in marine organisms that contain a variety of PLs with ether groups as well as structures resembling the diacylglycerol phospholipid.

In this study, the contents of PLs (PtdEtn, PtdCho, and SM) and plasmalogens (PlsEtn and PlsCho) in dried mussel, sea cucumber, big head shrimp, and opossum shrimp were analyzed through ^31^P-NMR. The compositions of fatty acids in PlsCho, which is a plasmalogen, and PtdCho, which is a diacylglycerol phospholipid with a choline group, were compared.

## 2. Results and Discussion

**Figure 2:**
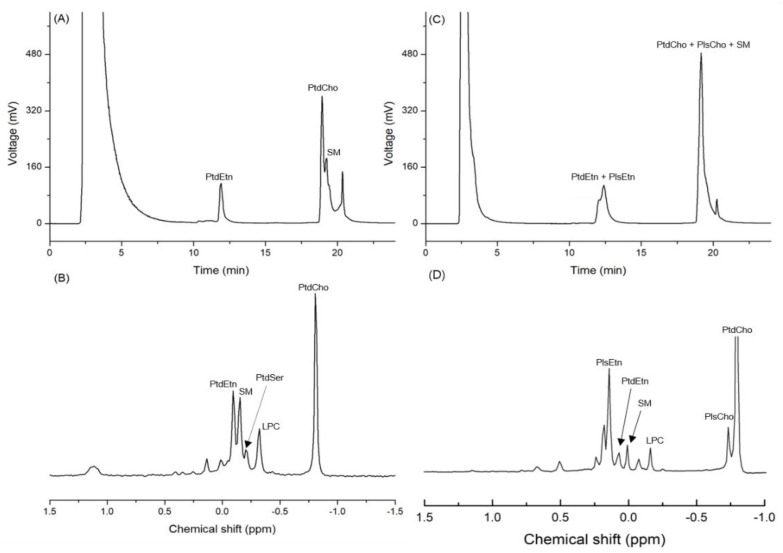
Chromatogram of phospholipids from infant formula (IF) and dried big head shrimp analyzed using HPLC-ELSD along with the ^31^P-NMR spectrum. (**A**) HPLC chromatogram of IF; (**B**) ^31^P-NMR spectrum of IF; (**C**) HPLC chromatogram of the dried big head shrimp; (**D**) ^31^P-NMR spectrum of the dried big head shrimp.

Based on the standard spiking, the three peaks detected on the chromatogram obtained by the HPLC-ELSD analysis of infant formula (IF) were identified as PtdEtn, PtdCho, and SM. The mean contents of PtdCho, PtdEtn, and SM in IF were 147.7, 104.2, and 76.4 mg/100 g of sample, respectively (Table 1 and Figure 2A). The ^31^P-NMR analysis of the same samples showed that the mean contents of PtdCho, PtdEtn, and SM were 136.3, 80.8, and 68.8 mg/100 g of sample, respectively (Table 1 and Figure 2B). The RSD (%) of the quantified values from the two analytical devices varied from 5.7 to 17.9, which is an acceptable range of values (Table 1). In the case of big head shrimp, however, the same HPLC-ELSD method was not used for measuring the content of PLs due to the co-elution (Table 1 and Figure 2C), while the quantitative analysis of ^31^P-NMR showed the contents of PtdCho, PtdEtn, and SM as 1677.9, 169.3, and 147.2 mg/100 g, respectively (Table 1 and Figure 2D). 

The column used in this study was a PVA-Sil column because the PVA coating can eliminate the unnecessary electrostatic interactions between the base organic compound and silanol group (-Si-O-H), whereas the bare silica may show tailing or irreversible adsorption effects of the strong electrostatic interactions between the base organic compound and silica [7]. In addition, the level of isolation of acidic organic compounds is low in the bare silica column but high in the PVA-Sil column; therefore, the latter is widely used for the analysis of PLs [8]. Nevertheless, the quantified values of PLs extracted from big head shrimp through HPLC-ELSD were not obtained due to the co-elution of diacyl phospholipid, SM, and plasmalogen. 

**Figure 3:**
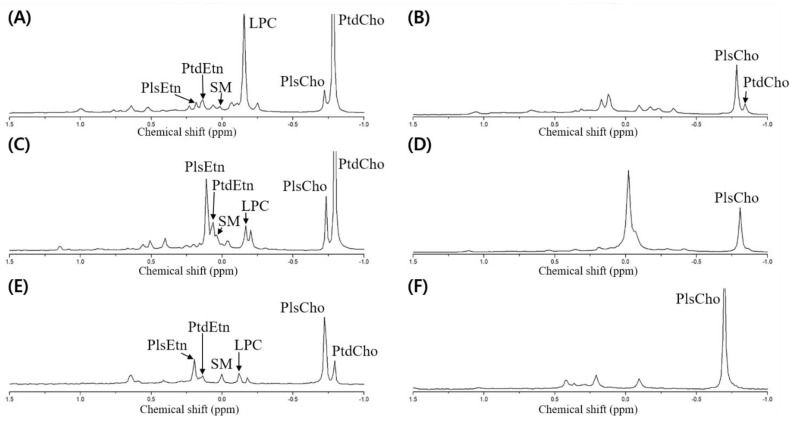
^31^P-NMR spectrum of phospholipid extracted from the dried opossum shrimp, mussel, and sea cucumber. (**A**) Dried opossum shrimp; (**B**) PLA_1_-pretreated dried opossum shrimp; (**C**) dried mussel; (**D**) PLA_1_-pretreated dried mussel; (**E**) dried sea cucumber; (**F**) PLA_1_-pretreated dried sea cucumber.

Meanwhile, the dried opossum shrimp, mussel, and sea cucumber were analyzed through ^31^P-NMR, followed by designation based on the standard spiking of PtdEtn, PtdCho, PtdSer, LPC, and SM (Figure 3A,C,E). Although no pretreatment was necessary to quantify the PLs by ^31^P-NMR, we verified the presence of plasmalogen after PLA_1_ pretreatment (Figure 3B,D,F). 

To detect the plasmalogen, an acid or PLA_1_ pretreatment is necessary [6,9]. In the case of the acid pretreatment, a HCl fume or a small amount of HCl is used, which allows rapid hydrolysis of the vinyl–ether linkage in plasmalogen to produce lyso-form and aldehyde, while the part linked with an acyl group is not hydrolyzed [10]. However, although only the vinyl groups should be hydrolyzed, the other acyl groups may be degraded upon acid treatment; therefore, PLA_1_ pretreatment was carried out to verify the presence of plasmalogen by ^31^P-NMR. 

The PLA_1_ pretreatment resulted in the hydrolysis of the acyl group at the *sn*-1 position of the diacyl phospholipid form (such as PtdEtn and PtdCho) to produce a lyso-form (LPE and LPC). As the reaction time was prolonged, the *sn*-2 acyl group could migrate to the *sn*-1 position to produce a glycerophospholipid form after further hydrolysis. When the resulting PLA_1_-treated samples were extracted by the Folch solvent, the PtdEtn and PtdCho components were collected as a hydrolyzed lyso-form whereas the plasmalogen was located without PLA_1_-mediated hydrolysis in the non-polar layer (chloroform). Therefore, plasmalogen along with a small amount of unreacted PtdCho was shown in the ^31^P-NMR spectrum (Figure 3B,D,F). In Figure 3, the spectrum is compared before and after the PLA_1_ pretreatment; a peak with a decrease in mol% indicates the diacylglycerol phospholipid form, whereas that with an increase in mol% indicates plasmalogen (PlsEtn and PlsCho).

Although HPLC-ELSD is useful for analyzing PL compositions, there is a possibility of co-elution in analyzing PLs in marine animals, especially those containing plasmalogen. This problem can be overcome by pretreatments such as PLA_1_ hydrolysis; however, it can prolong the analysis time. In addition, there is a possibility of a loss of PL components during the pretreatment process. In contrast, ^31^P-NMR allows the analysis of each PL type without pretreatment, as it can selectively detect the PLs. This is due to the variation in chemical shift with the difference in electron density around the ^31^P nucleus depending on the PL components [11]. Therefore, the subsequent quantitative analyses were performed using ^31^P-NMR.

The lipid contents (wt %) in dried big head shrimp, opossum shrimp, mussel, and sea cucumber were 8.5, 6.7, 10.1, and 1.2, respectively (Table 2), and the main PLs and plasmalogens were subsequently analyzed using ^31^P-NMR. 

Previously, the most abundantly found PL in marine animals was PtdCho [12], and similarly, in this study, the content of PtdCho was the highest (1603–1677.9 mg/100 g) in all the samples except sea cucumber.

In dried sea cucumber, the content of PtdCho (64.3 mg/100 g) was as low as approximately 1/26 of the content in big head shrimp (1677.9 mg/100 g). The weight percentage of PlsCho (36.4%) in the analyzed lipid weight was the highest among PLs, while the composition of PLs with choline (PtdCho + PlsCho) in sea cucumber was 47.9% and the composition of PLs with ethanolamine as the head group (PtdEtn + PlsEtn) was 11.4% (Table 2). In a previous study, where sea cucumber samples were purified with acetone and analyzed using HPLC-ELSD, the content of PtdCho among PLs was 55.1% and that of PtdEtn was 8.1% [13]. In another study using TOF-MS/MS [14], most of the PLs with ethanolamine as the head group (the ethanolamine class) were plasmalogens. The ^31^P-NMR analysis result in this study also showed that the content of PlsEtn was higher than that of PtdEtn. 

The ^31^P-NMR analysis results for dried mussel samples showed that the contents of PLs (mg/100 g) except PtdCho and LPC were the highest across the analyzed samples, as follows: PlsEtn (675.4), PtdEtn (345.1), PlsCho (379.0), and SM (198.8) (Table 2). Notably, similar to previous reports [14,15], the content of PlsEtn was higher than that of PtdEtn, showing 66.2% PlsEtn among the ethanolamine class (PtdEtn + PlsEtn).

The ^31^P-NMR analysis of dried big head shrimp and opossum shrimp showed that the content of PtdCho was the highest at 50.7 and 41.0% (*w*/*w* %) among PLs, respectively (Table 2), while it has been found that the content of PtdCho in Antarctic krill (*Euphausia superba*) oil is 66.5% (*w*/*w*) [16]. Due to the relatively high content of PlsEtn (19.1%), the lipids extracted from big head shrimp are likely to serve as a source of PlsEtn with the reported alleviating effect on Alzheimer’s disease [17].

**Figure 4:**
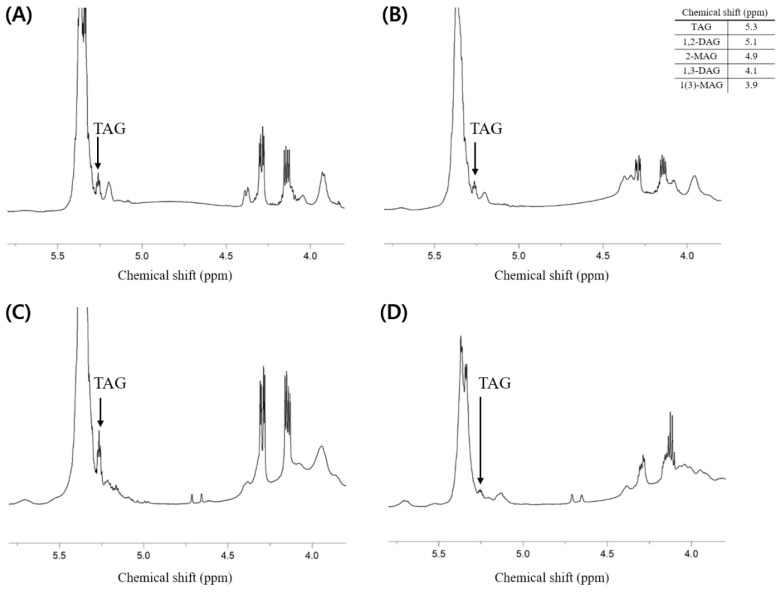
^1^H-NMR spectrum of total lipid extracted from the dried big head shrimp, opossum shrimp, mussel, and sea cucumber. (**A**) Dried big head shrimp; (**B**) Dried opossum shrimp; (**C**) Dried mussel; (**D**) Dried sea cucumber.

Additionally, the composition of neutral lipids was investigated. It is thought that the lipids extracted from each sample contain fewer neutral lipids than phospholipids because the total weight percent of phospholipids (i.e., PLs (*w*/*w* %)) presented in Table 2 is much higher than the weight percent of others containing neutral lipids. As a result of analysis through ^1^H-NMR, the component of neutral lipids that showed a clear peak and could confirm the presence was triacylglycerol (TAG), while it was difficult to identify the peaks corresponding to di- and mono-acylglycerol (Figure 4).

The main fatty acids at the *sn*-2 position in plasmalogens were reported. However, this is a very difficult task and there are a few things to consider, which are presented below. The first is the methylation method. In plasmalogens, the vinyl–ether linkage at the *sn*-1 position is rapidly hydrolyzed to form an aldehyde under acidic conditions, whereas under basic conditions, only the ester linkage at the *sn*-2 position, and not the vinyl–ether linkage, is known to be hydrolyzed [18]. The next one is obtaining the minimum amount of samples required for analysis. For example, the Folch extraction was performed on 0.5 g of mussel, and 50 mg of lipids was obtained, which corresponded to approximately 10% of the sample weight. The subsequent SPE isolated 16.5 mg of PLs, which corresponded to approximately 3.3% of the sample weight. Thereafter, to isolate PlsCho and PlsEtn, their concentrations were diluted to a level allowing the isolation, and after several repetitions of TLC, a small amount of approximately 3 mg of PlsCho and PlsEtn was obtained. This posed a challenge in determining the quantified values. In this study, to analyze the main fatty acids with the highest contents, the relative index (RI) obtained by dividing the area of fatty acids by the area of a fatty acid with the highest level was used in addition to the area percentage. Lastly, marine organisms contain unusual fatty acids such as the non-methylene-interrupted (NMI) form [19], and the commercial availability of these analytical standards is very limited.

The fatty acids at the *sn*-2 position of PlsCho in the four analyzed samples are presented in Table 3. When using a Supelco 37 Component FAME Mix as a standard, unidentifiable fatty acids (e.g., NMI fatty acids and those indicated as *unknowns*) ranged from 43% (opossum shrimp) to 87% (mussel). Among the identifiable fatty acids, EPA displayed the highest content in big head shrimp and sea cucumber, while in opossum shrimp and mussel samples, the highest contents were 10.8% palmitoleic acid (C16:1, n-7) and 7.5% docosadienoic acid, respectively. It has been found that palmitoleic acid is converted from palmitic acid through the action of Δ9-desaturase in the general lipid biosynthesis, while it may be subsequently converted to vaccenic acid through the action of elongase. According to the pathway, palmitic acid (RI = 0.55) and vaccenic acid (RI = 0.41) were also the main fatty acids of PlsCho in opossum shrimp along with docosadienoic acid (RI = 0.89), oleic acid (RI = 0.79), and eicosapentaenoic acid (RI = 0.55). A previous study reported that among the fatty acids of PlsCho in mussel, the content of palmitic acid was higher than that of others, as with eicosapentaenoic acid and docosahexaenoic acid [15]. 

In mussel, in this study, docosahexaenoic acid (RI = 0.54) was among the main fatty acids along with docosadienoic acid (RI = 1) and eicosapentaenoic acid (RI = 0.87). In addition, palmitic acid (RI = 0.55) and other saturated fatty acids including myristic acid (RI = 0.45) and stearic acid (RI = 0.52) were found. In the case of sea cucumber, the main fatty acids were eicosapentaenoic acid (RI = 1), docosadienoic acid (RI = 0.52), and arachidonic acid (RI = 0.4). Notably, in sea cucumber and opossum shrimp, nervonic acid (C24:1) was detected in a range of 0.8–1.1% at the *sn*-2 position of PlsCho. Nervonic acid is an unsaturated fatty acid required for brain growth and maintenance with a possible association with the control of cytosolic Ca^2+^ [20].

The fatty acid composition varied between PtdCho and PlsCho. Notably, for the four analyzed marine animals, the proportion of unidentifiable fatty acids varied from 11.0% (opossum shrimp) to 20.1% (sea cucumber) in PtdCho, which is much lower than that in PlsCho. In all samples except sea cucumber, palmitic acid was the most abundant fatty acid in PtdCho, while eicosapentaenoic acid was the most abundant one in sea cucumber (Table 4). 

In opossum shrimp and big head shrimp, eicosapentaenoic acid (RI = 0.51–0.54) and docosahexaenoic acid (RI = 0.38–0.39) were the other main fatty acids. For big head shrimp, in particular, the contents of oleic acid (RI = 0.77) and palmitoleic acid (RI = 0.61) were higher than those for the other three samples. In the case of mussel, docosahexaenoic acid (RI = 0.28), stearic acid (RI = 0.16), palmitoleic acid (RI = 0.16), and vaccenic acid (RI= 0.13) were found along with eicosapentaenoic acid (RI = 0.58) (Table 4).

According to the compositional results of total fatty acids of the lipid extracted from each sample (Table 5), palmitic acid was the highest (RI = 1) in big head shrimp, opossum shrimp, and mussel at 14.3, 21.9, and 18.6%, respectively. Unlike other samples, however, sea cucumber contained a small amount of palmitic acid (C16:0) at 2.3%, and the most abundant fatty acid (RI = 1) was eicosapentaenoic acid (26.0%) along with 12.1% docosadienoic acid and 9.8% arachidonic acid, which is consistent with the previous result [21] showing that sea cucumber contains a large amount of eicosapentaenoic acid and arachidonic acid. 

In addition, according to the results of other previous studies [22,23], palmitic acid, eicosapentaenoic acid, and docosahexaenoic acid are the main components of total fatty acids in shrimp and mussels. In Table 5, along with palmitic acid (RI = 1), eicosapentaenoic acid showed a high content of 10.4% (big head shrimp), 13.2% (opossum shrimp), and 13.2% (mussel). Meanwhile, all samples contained a large amount of other PUFAs as well as EPA. For example, big head shrimp, opossum shrimp, and mussel contained 11.1%, 14.0%, and 8.0% docosahexaenoic acid, respectively, while docosadienoic acid was found to be particularly high at 12.1% in the case of sea cucumber. 

Therefore, palmitic acid was not only the most abundant fatty acid among the total fatty acid composition of big head shrimp, opossum shrimp, and mussel but also the most abundant fatty acid in PtdCho of those three samples, which is thought to be reflected in total lipid composition. As mentioned earlier, PtdCho accounts for 32.6–50.7% of the extracted lipids from those three samples (Table 2, Table 4 and Table 5). In addition, the most abundant fatty acid in the total fatty acid composition of sea cucumber was eicosapentaenoic acid, which is consistent with the fatty acid contained in PlsCho, which accounts for 36.4% of the extracted lipid (Table 2, Table 3 and Table 5).

The main feed of marine animals, such as algae, can synthesize PUFAs, and through its ingestion, marine animals can increase the content of PUFAs. Sea cucumbers that ingested algae with enrichment of PUFAs are reported to show a higher content of PUFAs, such as eicosapentaenoic acid, compared to those that ingested corn meal or soybean meal [24]. Meanwhile, marine animals exhibiting a high content of PUFAs could have bacteria that can synthesize PUFAs in their intestines through ingestion [25]. In addition, the PUFA synthesis through the polyketide synthase (PKS) pathway in the intestines of invertebrates has been reported [26]. In addition, the variation in fatty acids found across PLs may be attributed to the seasonal fluctuations in the ecological environment. For example, the content of palmitic acid among PLs in mussels tended to be low in the season of low water temperature [27]. Meanwhile, the reason for PUFAs being the main fatty acids at the *sn*-2 position of plasmalogen could be found in the process of biosynthesis. Plasmalogen biosynthesis begins at dihydroxyacetone phosphate (DHAP), wherein the fatty acid at the *sn*-1 position is converted to fatty alcohol to form ether through the action of alkyl-DHAP synthase, and the ketone at the *sn*-2 position is removed by the acyl/alkyl-DHAP reductase to form the acyl linkage of fatty acid through the action of glycerophosphate acyltransferase (AAGPAT). Herein, due to the preference of AAGPAT3 on the PUFA species as acyl donors, the *sn*-2 position in 1-alkyl-2-acyl-glycerol 3-phosphate is bound with PUFAs with higher selectivity [28,29].

The qualitative and quantitative analyses of PLs demand a high level of sophistication as PLs have varying types; most of them are found in a trace quantity in the extracted lipids with a diversity of fatty acids engaged in ester linkages at the *sn*-1 and sn-2 positions that exhibit similar structures with similar physicochemical properties. For marine animals, in particular, for the ether PLs, their properties should be understood and suitable methods should be applied for analyses. In addition, the obtained values should not greatly deviate, irrespective of the analytic method, pretreatment, and device used. As mentioned in this study as well, the use of two different devices led to similar quantified values in one sample (i.e., the infant formula) but the presence of ether PLs such as plasmalogens would result in co-elution, resulting in difficulty of quantification. In such cases, simultaneous isolation is extremely challenging, unless there is a combination of successive HPLC separations with different analysis conditions. Moreover, the acquisition of internal standards is highly limited for the quantitative analysis of mass spectrometry. Alternatively, ^31^P-NMR may be suitable when the analysis conditions are secured to minimize the variation in chemical shifts. However, the limit of quantification value is considerably high in ^31^P-NMR compared to that in HPLC, so the PL concentration should be increased via such methods as solid phase extraction to allow the analysis.

## 3. Materials and Methods

### 3.1. Materials

The samples used were dried big head shrimp (*Solenocera melantho*, Tongyeong, Korea), opossum shrimp (*Neomysis awatschensis,* Tongyeong, Korea), mussel (*Mytilus galloprovincialis,* Tongyeong, Korea), and sea cucumber (*Apostichopus japonicus*, Seoul, Korea). The samples were purchased from a local market. Methanol, chloroform, isopropanol, hexane, water, and iso-octane, all of which were of HPLC grade, were obtained from Fisher Scientific Korea, Ltd. (Seoul, Korea). Anhydrous sodium sulfate (Na_2_SO_4_) and sodium hydroxide (NaOH) were purchased from Junsei Chemical Co., Ltd. (Tokyo, Japan) and DaeJung Chemicals & Metals (Siheung, Korea), respectively. Triphenyl phosphate (OP(OC_6_H_5_)_3_, TPP ≥ 99%), chloroform-d (CDCl_3_), and ethylenediaminetetraacetic acid (C_10_H_16_N_2_O_8_, EDTA), as well as the reference substances, L-α-Phosphatidylethanolamine (P7943-25MG), L-α-Phosphatidylcholine (P3556-25MG), L-α-Lysophosphatidylcholine (62962-50MG), L-α-Phosphatidyl-L-serine (P0474-25MG), and Sphingomyelin (85615-50MG), were purchased from Sigma-Aldrich (Seoul, Korea).

### 3.2. Lipid Extraction

The dried marine animal samples were blended and homogenized, and the lipids were extracted using the Folch method [30]. To a 0.5 g sample, 6 mL of distilled water and 24 mL of the Folch solvent (CHCl_3_: MeOH = 2:1, *v*/*v*) were added. The mixture was vortexed for 2 min and centrifuged at 3000 rpm for 10 min to produce the upper and lower phases. Using a glass pipette, the lower phase was filtered through an anhydrous sodium sulfate column. To the remaining upper phase, 12 mL of CHCl_3_ and 1 mL of MeOH were added, and the lower phase was obtained, as described previously. For the collected lower phase, the solvent was removed using nitrogen gas to obtain the lipids, whose contents were subsequently estimated.

### 3.3. Hydrolysis with Phospholipase A_1_

To the lipids extracted using the Folch method (50 mg), 24.5 mL of Tris-HCl buffer (pH 7.6), 6.125 mL of bile salt solution, 2.45 mL of 2.2% CaCl_2_ solution, and 0.5 mL of Phospholipase A_1_ (PLA_1_, Lecitase Ultra, Novozyme, Bagsvaerd, Denmark) were added. The mixture was vortexed for 2 min and then left to react in a shaking water bath (Vision scientific, Daejeon, Korea) at 38 °C and 100 rpm for 2 h. After the reaction, 24 mL of the Folch solvent was added, and the solution was vortexed for 2 min and centrifuged at 3000 rpm. The lower phase was filtered through an anhydrous sodium sulfate column, and the remaining upper phase was mixed with 12 mL of CHCl_3_ and 1 mL of MeOH to be vortexed for 2 min and centrifuged at 3000 rpm. The resulting lower phase was filtered through an anhydrous sodium sulfate column, and the solvent was removed using nitrogen gas to obtain the phospholipase A_1_-pretreated lipid.

### 3.4. Phospholipid Analysis Using HPLC-ELSD

HPLC was used to analyze the PLs in the infant formula and dried big head shrimp [7]. The HPLC device used was the SP930D (Yonglin, Yangyang, Korea). The column was the YMC-Pack PVA-Sil column (S-5 μm, 12 nm, 250 × 4.6 mm l.D., YMC, Kyoto, Japan), and for the detector, the ZAM-3000 (Schambeck SFD, Bad Honnef, Germany) was used as the ELSD. The ELSD evaporation temperature and pressure were set to 65 °C and 2.0, respectively.

The solvent system consisted of solvent A (isopropanol), solvent B (hexane), and solvent C (water). The gradient mode was used for the isolation, with the flow of 58% solvent A and 42% solvent B for 4 min from the onset, followed by the flow of 58% solvent A, 40% solvent B, and 2% solvent C for 7 min. Finally, the condition of 52% solvent A, 40% solvent B, and 8% solvent C was applied to the flow for 13 min. The total analysis time was 24 min. Each sample was dissolved in the Folch solvent for a 20 μL injection.

### 3.5. Phospholipid Analysis Using ^31^P-NMR and Neutral Lipid Analysis Using ^1^H-NMR

The extracted lipids were mixed with 1 mL of TPP solution (7.0 mg/10 mL dissolved in CDCl_3_), 1 mL MeOH, and 1 mL EDTA-Na^+^ solution (0.2 M, pH = 7.2). The mixture was vortexed for 2 min and centrifuged at 3000 rpm for 10 min. The isolated lower phase was filtered through an anhydrous sodium sulfate column and then placed in an NMR tube. The NMR device used was the Bruker Avance III-600 spectrometer (Bruker BioSpin, Billerica, MA, USA). When analyzing samples, the inverse gating decoupling was used to suppress the nuclear Overhauser effect. The ^31^P-NMR conditions were as follows: Probe temperature, 25 °C; excitation pulse, 30°; number of data points, 64 K; relaxation delay, 2 s; pulse width, 11.05 μs; acquisition time, 0.34 s; and number of scans, 256.

For quantitative analysis, the equations used in a previous study were applied after modifications [31]:

(1) $$\mathrm{PL}\;(µ\mathrm{mol}/100\;g\;\mathrm{of}\;\mathrm{sample})=\frac{I_{\left(PL\right)}\times A\times 100}{I_{\left(TPP\right)}\times m}$$

where *I*_(*PL*)_ indicates the peak area of PLs, *A* indicates the TPP (µmol) added as the internal standard (IS), *I*_(*TPP*)_ indicates the peak area of the added TPP, and *m* indicates the amount of sample used in the analysis. The molecular weights for calculation were as follows: PtdCho, 775.3539; PlsCho, 758.3469; LPC, 516.2883; SM, 739.6923; PtdEtn, 732.2649; PlsEtn, 715.2579. ^1^H-NMR (Bruker Avance III-600 spectrometer, Bruker BioSpin, Billerica, MA, USA) was used to determine the neutral lipid composition. After dissolving 50 mg of a sample in 700 μL of chloroform-d, it was taken into an NMR tube and analyzed. Analysis conditions were set to acquisition time 2.656 s, spectral width 12,335.5 Hz, and 16 scans, and chemical shifts (δ) were obtained by setting TMS to δ = 0 ppm.

### 3.6. Isolation of Phospholipids Using Solid Phase Extraction Column

To isolate the PLs from the extracted samples, a 6 mL LC-Si SPE column (LC-Si SPE, Supelco, Bellefonte, PA, USA) was used. Prior to sample loading, 6 mL of hexane was used in the conditioning of the cartridge. The phospholipase A_1_-pretreated lipids were dissolved in 500 µL CHCl_3_ and the entire amount was loaded. Next, neutral lipids such as triacylglycerol (TAG) were removed through the elution of 20 mL of the mixture (diethyl ether:hexane = 1:1, *v*/*v*). Following the elution using 20 mL MeOH for the cartridge, the solvent was removed to obtain the isolated PLs [7].

### 3.7. Thin-Layer Chromatography (TLC)

The phosphatidyl choline (PtdCho) and plasmalogen choline (PlsCho) for the fatty acid analysis were isolated through TLC. PtdCho was isolated from the lipids extracted using the Folch solvent. PlsCho was isolated from the lipids treated with phospholipase A_1_. After the sample loading, the TLC (silica gel 60 F_254_ glass plate, 20 × 20 cm, Merck, Darmstadt, Germany) was developed using a mixed solvent (chloroform:methanol:water, 75:25:3, *v*/*v*/*v*). Next, the plate was stained using a dye solution (0.05% primuline in acetone:water, 8:2, *v*/*v*) and detected at 365 nm.

### 3.8. Fatty Acid Analysis Using Gas Chromatography

To analyze the fatty acid composition at the *sn*-1 and *sn*-2 positions of PtdCho and at the *sn*-2 position of PlsCho, the bands isolated from the TLC were scrapped and methylation was carried out. For analyzing total fatty acid composition, lipids were extracted from each sample using Folch solvent followed by methylation. The analysis was performed using gas chromatography (GC, Agilent 6890 series, Santa Clara, CA, USA) with an SP-2560 column (i.d.: 100 m × 0.25 mm; film thickness: 0.2 µm; Supelco, Bellefonte, PA, USA). For the methylation, the scrapped bands were mixed with 1.5 mL of 0.5 N methanolic NaOH and vortexed for 30 s, and then the mixture was left to react in a water bath (85 °C) for 10 min. For extracting FAME, 1 mL of iso-octane and 1 mL of saturated NaCl were added. The mixture was vortexed for 1 min and centrifuged at 2500 rpm for 3 min. The upper phase was taken and filtered through an anhydrous sodium sulfate column to produce FAME, 1 µL of which was injected into the GC. The oven temperature was maintained at 100 °C for 4 min and then increased by 3 °C per min up to 240 °C, which was then maintained for 17 min. The injector temperature and the FID detector front temperature were set to 225 and 285 °C, respectively. The retention time of each fatty acid was compared against the reference substance, Supelco 37 Component FAME Mix (Sigma-Aldrich, St. Louis, MO, USA) [32].

## Figures and Tables

**Table 1:** Quantitative value (mg/100 g of sample) of phospholipids from infant formula and the dried big head shrimp as determined by HPLC-ELSD and ^31^P-NMR.

PL Concentration (mg/100 g of Sample) ^1)^						
Infant Formula				Dried Big Head Shrimp		
PLs	HPLC-ELSD	^31^P-NMR	RSD ^2)^	PLs	HPLC-ELSD	^31^P-NMR
PtdCho	147.7 ± 6.0 ^3)^	136.3 ± 1.1	5.7	PtdCho	Not measurable	1677.9 ± 273.6
SM	76.4 ± 7.8	68.8 ± 5.0	7.4	SM	Not measurable	147.2 ± 15.2
PtdEtn	104.2 ± 4.3	80.8 ± 13.8	17.9	PtdEtn	Not measurable	169.3 ± 20.7

^1)^ Analyzed from HPLC-ELSD and ^31^P-NMR expressed as the mean ± SD (*n* = 2). ^2)^ Relative standard deviation (RSD, %) = $\frac{Standard\;deviation}{mean}$ × 100. ^3)^ Quantitative values of each phospholipid using HPLC-ELSD and ^31^P-NMR, respectively. Values of infant formula were statistically analyzed by *t*-test. In the case of infant formula, there was no significant difference between the analyzed values of HPLC-ELSD and ^31^P-NMR. However, in the case of the dried big head shrimp, each value of HPLC-ELSD was not measurable due to the co-elution.

**Table 2:** Total lipid content (wt %) and quantitative value (mg/100 g) and composition (*w*/*w* %) of phospholipids extracted from the dried big head shrimp, opossum shrimp, mussel, and sea cucumber determined using ^31^P-NMR.

PLs (mg/100 g of Sample) ^1)^	Mussel	Sea Cucumber	Big Head Shrimp	Opossum Shrimp
	*Mytilus galloprovincialis*	*Apostichopus japonicus*	*Solenocera melantho*	*Neomysis awatschensis*
PtdCho	1661.6 ± 63.6	64.3 ± 25.5	1677.9 ± 273.6	1603.0 ± 186.8
PlsCho	379.0 ± 16.8	206.9 ± 88.5	262.3 ± 43.0	245.6 ± 29.4
LPC	190.3 ± 12.1	37.1 ± 14.4	67.6 ± 7.9	683.7 ± 42.1
SM	198.8 ± 9.5	26.8 ± 13.2	147.2 ± 15.2	102.2 ± 11.8
PtdEtn	345.1 ± 3.0	17.6 ± 9.4	169.3 ± 20.7	121.2 ± 17.8
PlsEtn	675.4 ± 7.7	51.5 ± 27.9	629.5 ± 53.4	217.9 ± 27.9
Total lipid content (wt %)	10.1 ± 0.4	1.2 ± 0.4	8.5 ± 0.2	6.7 ± 0.4
**PLs (*w*/*w* %) ^2)^**	**Mussel**	**Sea Cucumber**	**Big Head Shrimp**	**Opossum Shrimp**
	*Mytilus galloprovincialis*	*Apostichopus japonicus*	*Solenocera melantho*	*Neomysis awatschensis*
PtdCho	32.6 ± 0.2	11.5 ± 0.5	50.7 ± 2.6	41.0 ± 0.8
PlsCho	7.4 ± 0.1	36.4 ± 0.2	8.1 ± 2.2	6.3 ± 0.1
LPC	3.7 ± 0.1	6.7 ± 0.3	2.1 ± 0.1	17.6 ± 0.6
SM	3.9 ± 0.1	4.6 ± 0.4	4.5 ± 0.1	2.6 ± 0.0
PtdEtn	6.8 ± 0.2	2.9 ± 0.4	5.2 ± 0.1	3.1 ± 0.1
PlsEtn	13.3 ± 0.6	8.5 ± 1.2	19.1 ± 0.5	5.6 ± 1.3
Others ^3)^ (e.g., neutral lipid, unknowns)	32.3	29.4	10.3	23.8

^1)^ Results are obtained from ^31^P-NMR analysis and expressed as the mean ± SD (*n* = 2). PL (mg/100 g of sample) = $\frac{I_{\left(PL\right)}\times \;A\;\times \;100\;\times \;Phospholipid\;molecular\;weight}{I_{\left(TPP\right)}\times \;m}$. I_(PL)_, I_(TPP)_, A, and m are described in the section on phospholipid analysis using ^31^P-NMR in Section 3. ^2)^ PL (*w*/*w* %) = $\frac{Weight\;of\;each\;phospholipid}{Weight\;of\;analysed\;extracted\;lipid}\;\times \;100$. ^3)^ Others (*w*/*w* %) = $\left(1-\frac{Weight\;of\;each\;phospholipid}{Weight\;of\;analysed\;extracted\;lipid}\right)\;\times \;100$.

**Table 3:** Fatty acid composition (area %) and relative index of plasmalogen in the extracted lipids from the dried big head shrimp, opossum shrimp, mussel, and sea cucumber. Relative index = value of each fatty acid area/value of the largest known fatty acid area.

PlsCho				
Fatty Acids	Big Head Shrimp	Opossum Shrimp	Mussel	Sea Cucumber
	*Solenocera melantho*	*Neomysis awatschensis*	*Mytilus galloprovincialis*	*Apostichopus japonicus*
Myristic acid (C14:0)	0.11 ± 0.01 (1.2%)	0.09 ± 0.00 (0.9%)	0.45 ± 0.05 (3.4%)	0.12 ± 0.00 (2.5%)
Palmitic acid (C16:0)	0.12 ± 0.00 (1.3%)	0.55 ± 0.05 (5.9%)	0.55 ± 0.01 (4.1%)	0.05 ± 0.00 (1.0%)
Palmitoleic acid (C16:1, n-7)	0.12 ± 0.00 (1.3%)	1.00 ± 0.00 (10.8%)	ND	ND
Stearic acid (C18:0)	0.10 ± 0.01 (1.1%)	0.30 ± 0.04 (3.2%)	0.52 ± 0.05 (3.9%)	0.06 ± 0.00 (1.1%)
Oleic acid (C18:1, n-9)	0.87 ± 0.09 (9.4%)	0.79 ± 0.07 (8.6%)	0.27 ± 0.01 (2.1%)	0.08 ± 0.01 (1.6%)
Vaccenic acid (C18:1, n-7)	0.31 ± 0.01 (3.4%)	0.41 ± 0.04 (4.5%)	0.17 ± 0.01 (1.3%)	0.17 ± 0.00 (3.5%)
Linoleic acid (C18:2, n-6)	ND	0.10 ± 0.00 (1.0%)	0.14 ± 0.00 (1.1%)	ND
Arachidic acid (C20:0)	ND	ND	ND	ND
cis-11-Eicosenoic acid (C20:1, n-9)	ND	0.09 ± 0.00 (0.9%)	0.13 ± 0.01 (1.0%)	0.02 ± 0.00 (0.5%)
α-Linolenic acid (C18:3, n-3)	ND	0.07 ± 0.00 (0.7%)	ND	ND
Eicosadienoic acid (C20:2, n-6)	0.24 ± 0.01 (2.6%)	0.07 ± 0.00 (0.8%)	ND	0.02 ± 0.00 (4.1%)
Behenic acid (C22:0)	ND	0.04 ± 0.00 (0.5%)	ND	ND
cis-8, 11, 14-Eicosatrienoic acid (C20:3, n-6)	ND	0.04 ± 0.00 (0.4%)	ND	ND
Erucic acid (C22:1, n-9)	0.22 ± 0.00 (2.4%)	0.04 ± 0.01 (0.5%)	ND	ND
Arachidonic acid (AA, C20:4, n-6)	0.26 ± 0.01 (2.8%)	0.16 ± 0.00 (1.8%)	0.18 ± 0.00 (1.3%)	0.40 ± 0.00 (8.3%)
Docosadienoic acid (C22:2, n-6)	0.61 ± 0.05 (6.6%)	0.89 ± 0.02 (9.6%)	1.00 ± 0.00 (7.5%)	0.52 ± 0.00 (10.7%)
Lignoceric acid (C24:0)	ND	0.05 ± 0.01 (0.6%)	ND	ND
Eicosapentaenoic acid (EPA, C20:5, n-3)	1.00 ± 0.00 (10.9%)	0.55 ± 0.04 (6.0%)	0.87 ± 0.06 (6.6%)	1.00 ± 0.00 (20.5%)
Nervonic acid (C24:1, n-9)	ND	0.08 ± 0.01 (0.8%)	ND	0.05 ± 0.00 (1.1%)
Docosapentaenoic acid (DPA, C22:5, n-3)	0.77 ± 0.03 (8.4%)	ND	0.13 ± 0.00 (1.0%)	ND
Docosahexaenoic acid (DHA, C22:6, n-3)	0.40 ± 0.02 (4.4%)	0.32 ± 0.01 (3.5%)	0.54 ± 0.03 (4.0%)	ND
Unknowns	5.03 ± 0.33 (54.7%)	3.98 ± 0.12 (43.0%)	11.42 ± 0.48 (87.0%)	2.57 ± 0.10 (52.7%)

**Table 4:** Fatty acid composition (area %) and relative index of phosphatidylcholine in the extracted lipids from the dried big head shrimp, opossum shrimp, mussel, and sea cucumber. Relative index = value of each fatty acid area/value of the largest known fatty acid area.

PtdCho				
Fatty Acid	Big Head Shrimp	Opossum Shrimp	Mussel	Sea Cucumber
	*Solenocera melantho*	*Neomysis awatschensis*	*Mytilus galloprovincialis*	*Apostichopus japonicus*
Myristic acid (C14:0)	ND	0.12 ± 0.00 (3.5%)	0.10 ± 0.00 (2.9%)	ND
Palmitic acid (C16:0)	1.00 ± 0.00 (21.0%)	1.00 ± 0.00 (28.3%)	1.00 ± 0.00 (30.7%)	0.25 ± 0.01 (7.7%)
Palmitoleic acid (C16:1, n-7)	0.61 ± 0.01 (12.7%)	0.17 ± 0.00 (4.7%)	0.16 ± 0.00 (4.8%)	ND
cis-10-Hetadeccenoic acid (C17:1)	0.10 ± 0.00 (2.0%)	ND	ND	ND
Stearic acid (C18:0)	0.22 ± 0.01 (4.6%)	0.15 ± 0.00 (4.2%)	0.16 ± 0.00 (4.8%)	0.35 ± 0.02 (10.7%)
Oleic acid (C18:1, n-9)	0.77 ± 0.02 (16.2%)	0.35 ± 0.00 (9.8%)	0.06 ± 0.00 (1.8%)	0.17 ± 0.01 (5.3%)
Vaccenic acid (C18:1, n-7)	0.32 ± 0.01 (6.7%)	0.05 ± 0.00 (2.6%)	0.13 ± 0.00 (4.1%)	0.13 ± 0.00 (4.0%)
Linoleic acid (C18:2, n-6)	0.10 ± 0.00 (2.1%)	0.08 ± 0.00 (2.1%)	0.04 ± 0.00 (1.1%)	ND
Arachidic acid (C20:0)	ND	0.02 ± 0.00 (0.5%)	ND	ND
γ-Linolenic acid (C18:3, n-6)	0.03 ± 0.00 (0.7%)	0.05 ± 0.00 (1.4%)	0.06 ± 0.00 (1.8%)	0.26 ± 0.00 (7.9%)
cis-11-Eicosenoic acid (C20:1, n-9)	0.04 ± 0.00 (0.8%)	0.03 ± 0.00 (1.0%)	0.04 ± 0.00 (1.1%)	ND
α-Linolenic acid (C18:3, n-3)	0.03 ± 0.00 (0.6%)	0.08 ± 0.00 (2.1%)	0.03 ± 0.00 (0.9%)	ND
Eicosadienoic acid (C20:2, n-6)	0.03 ± 0.00 (0.7%)	ND	ND	ND
Erucic acid (C22:1, n-9)	ND	0.02 ± 0.00 (0.6%)	0.02 ± 0.00 (0.7%)	0.16 ± 0.00 (4.9%)
Arachidonic acid (AA, C20:4, n-6)	0.14 ± 0.00 (2.9%)	0.05 ± 0.00 (1.5%)	0.05 ± 0.00 (1.5%)	0.29 ± 0.01 (8.8%)
Docosadienoic acid (C22:2, n-6)	ND	0.02 ± 0.00 (0.5%)	ND	ND
Eicosapentaenoic acid (EPA, C20:5, n-3)	0.51 ± 0.01 (10.6%)	0.54 ± 0.00 (15.3%)	0.58 ± 0.01 (17.7%)	1.00 ± 0.01 (30.6%)
Docosapentaenoic acid (DPA, C22:5, n-3)	0.05 ± 0.00 (1.1%)	ND	ND	ND
Docosahexaenoic acid (DHA, C22:6, n-3)	0.39 ± 0.01 (8.3%)	0.38 ± 0.01 (10.8%)	0.28 ± 0.00 (8.5%)	ND
Unknowns	0.53 ± 0.00 (11.2%)	0.39 ± 0.01 (11.0%)	0.58 ± 0.01 (19.0%)	0.66 ± 0.01 (20.1%)

**Table 5:** Fatty acid composition (area %) and relative index of total lipids extracted from the dried big head shrimp, opossum shrimp, mussel, and sea cucumber. Relative index = value of each fatty acid area/value of the largest known fatty acid area.

Total Fatty Acid Composition				
Fatty Acid	Big Head Shrimp	Opossum Shrimp	Mussel	Sea Cucumber
	*Solenocera melantho*	*Neomysis awatschensis*	*Mytilus galloprovincialis*	*Apostichopus japonicus*
Butyric acid (C4:0)	ND	trace (0.0%)	trace (0.0%)	trace (0.0%)
Capric acid (C10:0)	trace (0.0%)	trace (0.0%)	ND	ND
Lauric acid (C12:0)	0.02 ± 0.00 (0.3%)	0.01 ± 0.00 (0.2%)	trace (0.0%)	trace (0.0%)
Myristic acid (C14:0)	0.19 ± 0.00 (2.7%)	0.30 ± 0.00 (6.7%)	0.25 ± 0.00 (4.6%)	0.05 ± 0.00 (1.4%)
Myristoleic acid (C14:1)	0.02 ± 0.00 (0.2%)	trace (0.0%)	trace (0.0%)	trace (0.0%)
Palmitic acid (C16:0)	1.00 ± 0.00 (14.3%)	1.00 ± 0.00 (21.9%)	1.00 ± 0.00 (18.6%)	0.09 ± 0.00 (2.3%)
Palmitoleic acid (C16:1, n-7)	0.88 ± 0.01 (12.5%)	0.30 ± 0.00 (6.6%)	0.55 ± 0.01 (10.2%)	0.14 ± 0.00 (3.7%)
Stearic acid (C18:0)	0.33 ± 0.00 (4.8%)	0.21 ± 0.00 (4.5%)	0.22 ± 0.00 (4.1%)	0.12 ± 0.00 (3.1%)
Oleic acid (C18:1, n-9)	0.80 ± 0.00 (11.4%)	0.27 ± 0.01 (5.9%)	0.15 ± 0.00 (2.9%)	0.05 ± 0.00 (1.2%)
Vaccenic acid (C18:1, n-7)	0.42 ± 0.00 (5.9%)	0.11 ± 0.00 (2.3%)	0.08 ± 0.12 (3.1%)	0.09 ± 0.00 (2.5%)
Linoleic acid (C18:2, n-6)	0.05 ± 0.00 (0.7%)	0.10 ± 0.00 (2.2%)	0.15 ± 0.00 (2.8%)	0.01 ± 0.00 (0.4%)
Arachidic acid (C20:0)	0.03 ± 0.00 (0.4%)	0.01 ± 0.00 (0.3%)	0.01 ± 0.00 (0.2%)	0.04 ± 0.00 (1.1%)
γ-Linolenic acid (C18:3, n-6)	trace (0.0%)	0.01 ± 0.00 (0.2%)	trace (0.0%)	trace (0.0%)
cis-11-Eicosenoic acid (C20:1, n-9)	0.07 ± 0.00 (1.0%)	0.03 ± 0.00 (0.6%)	0.13 ± 0.00 (2.5%)	0.02 ± 0.00 (0.5%)
α-Linolenic acid (C18:3, n-3)	0.09 ± 0.00 (1.3%)	0.09 ± 0.00 (1.9%)	0.14 ± 0.00 (2.6%)	0.03 ± 0.00 (0.7%)
Eicosadienoic acid (C20:2, n-6)	0.04 ± 0.00 (0.5%)	0.07 ± 0.09 (0.2%)	0.01 ± 0.00 (0.3%)	0.02 ± 0.00 (0.6%)
cis-8, 11, 14-Eicosatrienoic acid (C20:3, n-6)	0.01 ± 0.00 (0.1%)	trace (0.0%)	trace (0.0%)	trace (0.0%)
Erucic acid (C22:1, n-9)	0.01 ± 0.00 (0.1%)	ND	0.01 ± 0.00 (0.1%)	0.03 ± 0.00 (0.7%)
cis-11, 14, 17-Eicosatrienoic acid (C20:3, n-3)	ND	ND	0.01 ± 0.00 (0.1%)	0.08 ± 0.00 (2.0%)
Arachidonic acid (C20:4, n-6)	0.23 ± 0.00 (2.9%)	0.07 ± 0.00 (1.6%)	0.09 ± 0.00 (1.6%)	0.38 ± 0.00 (9.8%)
Docosadienoic acid (C22:2, n-6)	trace (0.0%)	ND	ND	0.47 ± 0.00 (12.1%)
Eicosapentaenoic acid (EPA, C20:5, n-3)	0.73 ± 0.00 (10.4%)	0.60 ± 0.00 (13.2%)	0.71 ± 0.01 (13.2%)	1.00 ± 0.00 (26.0%)
Nervonic acid (C24:1, n-9)	0.02 ± 0.00 (0.3%)	0.02 ± 0.00 (0.4%)	ND	0.04 ± 0.00 (0.9%)
Docosapentaenoic acid (DPA, C22:5, n-3)	0.07 ± 0.00 (1.1%)	0.02 ± 0.00 (0.5%)	0.05 ± 0.00 (1.0%)	0.01 ± 0.00 (0.2%)
Docosahexaenoic acid (DHA, C22:6, n-3)	0.78 ± 0.00 (11.1%)	0.64 ± 0.00 (14.0%)	0.43 ± 0.01 (8.0%)	0.02 ± 0.00 (0.4%)
Unknowns	1.24 ± 0.00 (17.7%)	0.80 ± 0.00 (17.6%)	1.28 ± 0.01 (23.8%)	1.16 ± 0.00 (30.1%)

## Data Availability

All relevant data are included in the article.

## Sample Availability

Samples of the compounds are not available from the authors.

## Abbreviations

PL	Phospholipid
PtdCho	Phosphatidyl choline
PtdEtn	Phosphatidyl ethanolamine
PtdSer	Phosphatidyl serine
PtdIns	Phosphatidyl inositol
PtdGro	Phosphatidyl glycerol
LPC	Lysophosphatidyl choline
PlsCho	Plasmalogen choline
PlsEtn	Plasmalogen ethanolamine
PLA_1_	Phospholipase A_1_
